# Incidence and Outcomes of Unstable Angina in Patients with Low High-Sensitivity Cardiac Troponin I Values—A Substudy of the RACE-IT Trial

**DOI:** 10.3390/jcm15093208

**Published:** 2026-04-23

**Authors:** Raef Fadel, Joseph Miller, Bernard Cook, Felix Nguyen, Mohammad Alqarqaz, Brittany Fuller, Mir Babar Basir, Tiberio Frisoli, Pedro Villablanca, Ahmad Jabri, Khaldoon Alaswad, Akshay Khandelwal, Natesh Lingam, Brian O’Neill, Henry Kim, Pedro Engel Gonzalez, Elizabeth Pielsticker, Gerald Koenig, Seth Krupp, Nicholas L. Mills, Simon Mahler, Phillip Levy, Benjamin Brennan, Shane Bole, Sachin Parikh, Khaled Nour, Michael Hudson, Bryan Zweig, Omr Abuzahrieh, Chaun Gondolfo, James McCord

**Affiliations:** 1 Henry Ford Heart and Vascular Institute, Detroit, MI 48202, USAjmccord1@hfhs.org (J.M.); 2 Henry Ford Hospital, Detroit, MI 48202, USA; 3 School of Medicine, Wayne State University, Detroit, MI 48201, USA; 4 Allegheny Health Network & Allegheny General Hospital, Pittsburgh, PA 15212, USA; 5 The Usher Institute, University of Edinburgh, Edinburgh EH16 4UX, UK; 6 School of Medicine, Wake Forest University, Winston-Salem, NC 27157, USA

**Keywords:** unstable angina, troponin, myocardial infarction

## Abstract

**Background:** Unstable angina has become an exceedingly rare diagnosis in the era of high-sensitivity cardiac troponin (hs-cTn). **Objectives:** We sought to identify the incidence of unstable angina and characterize patients with low hs-cTn in emergency departments (EDs). **Methods:** A prespecified secondary analysis of the Rapid Acute Coronary Syndrome Exclusion using high-sensitivity cardiac Troponin I (RACE-IT) trial was conducted. RACE-IT was a stepped-wedge randomized trial comparing two rule-out protocols (0/1- and 0/3 h) for myocardial infarction (MI) in nine EDs from July 2020 to April 2021. All patients had hs-cTnI (Beckman Coulter) concentrations below or equal to the 99th percentile upper reference limit of 18 ng/L. The primary outcome was unstable angina, based on the ISCHEMIA trial definition, which required electrocardiographic changes or findings at coronary angiography (angiographic evidence of plaque rupture or thrombus). **Results:** Of the 32,608 patients in the trial, 60 patients (0.2%) met the definition of unstable angina, of whom 46 (77%) had obstructive disease at coronary angiography and 17 (28%) had an ischemic electrocardiogram. Coronary revascularization was performed in 45 (75%) patients and seven (12%) had left main or 3-vessel coronary artery disease. There were seven (12%) patients with non-obstructive coronary artery disease, and seven (12%) who had angiographically unremarkable coronary arteries. Patients with unstable angina were older (*p* = 0.015), more likely to be male (*p* = 0.005), with a higher prevalence of hypertension (*p* < 0.001), known coronary artery disease (*p* < 0.001), peripheral vascular disease (*p* = 0.035), and a higher serum creatinine (*p* = 0.018). At 30 days, two patients had a type 1 MI and there were no deaths. **Conclusions:** Unstable angina was diagnosed in 1 in 500 patients with a low hs-cTnI value at presentation to the ED and these patients had an excellent prognosis at 30 days. These patients tend not to have high-risk anatomy and one in four had non-obstructive coronary artery disease or angiographically unremarkable coronary arteries.

## 1. Background

Utilization of high-sensitivity cardiac troponin (hs-cTn) for the rapid diagnosis or exclusion of myocardial infarction (MI) in the emergency department (ED) is the standard of care for the management of patients with possible MI and is recommended by guidelines [1,2]. Patients with symptoms of myocardial ischemia at risk for major adverse cardiac events (MACE) who do not meet the criteria for MI are often diagnosed as having unstable angina [3,4], but the diagnosis is more subjective and has been variably applied in clinical practice [5]. This is primarily due to the reliance on the clinical history for the diagnosis, despite the fact that symptoms of myocardial ischemia are heterogeneous [3].

The true incidence of unstable angina is unclear, especially since the adoption of cTn testing and particularly with the more recent use of hs-cTn assays. Many patients with unstable angina in prior studies utilizing creatine kinase (CK-MB) would have been reclassified as MI if a more sensitive cTn assay had been used [5]. The American College of Cardiology and American Heart Association guidelines for the management of patients with acute coronary syndrome recognize that close to a third of patients who had previously been diagnosed with unstable angina without CK-MB elevation have cTn elevation and would be reclassified as MI [6]. With the introduction of hs-cTn assays, the incidence of unstable angina has been reduced further [7].

The RACE-IT (Rapid Acute Coronary Syndrome Exclusion using high-sensitivity cardiac I Troponin) trial recently compared an accelerated protocol (AP) over 1 h to a standard of care (SC) protocol over 3 h using a hs-cTnI assay in the diagnosis and management of patients with possible MI [8]. In this prespecified secondary analysis of the trial we sought to evaluate the incidence of unstable angina and characterize these patients.

## 2. Methods

### 2.1. Study Design

The RACE-IT trial was a stepped-wedge, randomized controlled trial of consecutive patients evaluated for possible MI across the nine hospitals from Henry Ford Health in Detroit, Michigan from July 2020 to April 2021 (Clinicaltrials.gov registration identifier: NCT04488913). Details of this study have previously been published [8,9]. Patients were included if they had hs-cTnI measured using the Access Beckman Coulter high-sensitivity assay and an electrocardiogram (ECG) was obtained in the ED. Exclusion criteria included age less than 18 years, ST-segment elevation MI, trauma, transfers from other facilities, residence outside of Michigan, resident in hospice, and hs-cTnI above the 99th percentile upper reference limit (URL) of 18 ng/L on testing within the first 3 h from presentation to the ED. RACE-IT compared a 1 h protocol where all hs-cTnI values > 4 ng/L were reported to a 3 h protocol where only values ≥ 18 ng/L were reported. The assay has a limit of detection of 0.3 ng/L and a limit of quantification of 0.8 ng/L [10].

In this analysis, our aim was to identify and characterize patients with unstable angina and to determine their major adverse cardiac event (MACE) rate at 30 days. MACE was defined as subsequent MI or death from any cause at 30 days from presentation. All patients from the RACE-IT trial were included in this secondary analysis, which then stratified patients as having unstable angina or not.

### 2.2. Definitions and Data Adjudication

Unstable angina was defined according to the criteria used in the ISCHEMIA trial [11]. Unstable angina was considered in those with ischemic symptoms at rest, or an accelerating pattern that occurred at a lower activity threshold resulting in a visit to a healthcare facility, with cardiac troponin concentrations below the 99th percentile URL, and at least one of the following: (1) new or worsening ST-segment or T-wave changes on a resting electrocardiogram consistent with myocardial ischemia, or (2) angiographic evidence of a ruptured plaque or thrombus in an epicardial coronary artery thought to be responsible for the ischemic symptoms or signs. Obstructive coronary artery disease was defined as a stenosis ≥70% in a major epicardial vessel, ≥50% in the left main stem, or evidence of flow limitation by physiologic testing including fractional flow reserve or instantaneous wave-free ratio.

Cases were adjudicated by interventional cardiologists, general cardiologists, or cardiology fellows. All patients who had coronary angiograms were reviewed by interventional cardiologists to determine unstable angina. Cases of patients that did not have a coronary angiogram but had an elevation of hs-cTnI > 18 after admission, were reviewed by cardiology fellows to determine unstable angina. Outcomes at 30 days were identified by a review of the electronic medical record (Epic Systems). An interventional cardiologist adjudicated MIs in accordance with the universal definition of MI [12]. Twelve institutions in Michigan use Epic and participate in a health information exchange. Death at 30 days was determined by a review of the electronic medical record and by accessing the National Death Index. For deaths determined through the National Death Index, diagnostic codes consistent with a cardiac cause of death (120–125, 146, 149) were used to classify cardiac death [13]. All cases of cardiac death or MI at 30 days were reviewed by a general cardiologist to determine if the original presentation constituted unstable angina. Cardiology fellows also reviewed all the ECGs of patients who were adjudicated to have unstable angina to see if they met the ISCHEMIA trial definition of ischemic ECG changes.

### 2.3. Statistical Analysis

Demographic and comorbid variables in United States patients were compared to the corresponding variables in the non-unstable angina cohort using appropriate statistical tests for each variable type and sample size. All continuous variables were analyzed using the Wilcoxon Rank-Sum Test. Due to the small sample size, differences in peripheral vascular disease were assessed using Fisher’s Exact Test—all other categorical or binary variables were analyzed using a Chi-Squared Test. All analysis was performed using R version 4.4.0.

## 3. Results

### 3.1. Baseline Characteristics and Details of Presentation

Of the 32,608 patients enrolled in the RACE-IT trial, 60 patients (0.2%) met the definition of unstable angina, of whom 37 (62%) were enrolled from the group evaluated using the advanced protocol and 23 (38%) from the group evaluated using the standard care protocol. Patients with unstable angina were older (*p* = 0.032) and were more likely to be male (*p* = 0.012). There was a higher prevalence of hypertension (*p* < 0.001), previous coronary artery disease (*p* < 0.001), and peripheral vascular disease (*p* = 0.030) among patients with unstable angina. Additionally, patients with unstable angina had higher creatinine concentrations (*p* = 0.043) with a trend toward a lower estimated glomerular filtration rate (*p* = 0.067) (Table 1).

### 3.2. Diagnostic Results in Patients with Unstable Angina

In the overall study there were 400 coronary angiograms performed. All but two of the patients had their coronary angiograms during the index hospitalization, and all were performed within 30 days of the index presentation. Among those who underwent angiography, 46 (77%) patients had obstructive disease, and all but one underwent a revascularization procedure (41 percutaneous interventions and four coronary artery bypass surgeries). Of note, only seven (12%) had high-risk coronary disease (left main or 3-vessel CAD), seven (12%) had non-obstructive disease, and seven (12%) had angiographically unremarkable coronary arteries. All unstable angina patients with angiographically unremarkable coronary arteries or non-obstructive disease were noted to have ischemic ECG changes. Results of coronary angiography are shown in Table 2.

On presentation, 17 (28%) of the patients classified as having unstable angina following angiographic review had evidence of myocardial ischemia on the electrocardiogram including T-wave inversion (25%) and/or ST-segment depression (13%) (Table 3). Also, 12 patients (20%) in the unstable angina group had extremely low hs-cTnI values < 4 ng/L.

### 3.3. Outcomes

In patients not meeting the criteria for unstable angina, 26 (0.1%) experienced an MI and 64 (0.3%) died of any cause at 30 days with eight of these deaths attributed to a cardiac cause. There were no deaths at 30 days in patients classified as having unstable angina. However, there were two (3%) patients with a type 1 MI within 30 days. The first patient was an 86-year-old male with known coronary artery disease who presented with episodic chest pain at rest without myocardial ischemia on the electrocardiogram in whom all hs-cTnI concentrations were <18 ng/L. He was advised to stay for further evaluation, but he wanted to leave and follow up with his cardiologist. He presented five days later with an inferior ST-segment elevation MI and underwent PCI to the right coronary artery. The second patient was a 75-year-old male with known coronary artery disease who presented with chest pain similar to the symptoms he had experienced during a previous MI. He had no myocardial ischemia on the electrocardiogram and had serial hs-cTnI values of 6 and 7 ng/L. He had a nuclear stress test within the past year that showed a small area of ischemia in the left circumflex distribution, and he was discharged home. He returned two weeks later with a diagnosis of non-ST-segment elevation MI. He underwent PCI of the right coronary artery.

## 4. Discussion

In this prespecified secondary analysis of the RACE-IT trial we report the incidence of unstable angina in a large consecutive patient population evaluated for possible MI in whom serial hs-cTnI measurements were within the normal reference range at presentation. We report three major findings.

First, the incidence of unstable angina in patients with hs-cTnI values < 99th, using the definition from the ISCHEMIA trial, is very low, only 0.2% in our study. This is in line with prior studies which have shown that the incidence of unstable angina decreases with the introduction of hs-cTn assays. Wilson et al. reported similar findings when assessing patients enrolled in the randomized trial to evaluate the relative protection against Post-PCI Microvascular Dysfunction (PROTECT)-TIMI 30 trial. With the use of a hs-cTnI assay with a detection limit down to 0.2 ng/L and a 99th percentile reference limit of 3.0 ng/mL, compared to prior standard commercially available assays at the time, 44% of patients previously diagnosed with unstable angina demonstrated troponin levels at presentation exceeding the 99th percentile, which increased to 82% of patients by the 8 h mark [14].

Second, the prognosis at 30 days is excellent in patients with confirmed unstable angina. There were no patients who died and only two had an MI within 30 days. This could be explained by the low prevalence of high-risk coronary anatomy involving the left main stem or all three major epicardial coronary arteries. Other studies have reported similar findings. Gallone et al. reported that in 240 patients with unstable angina in whom hs-cTnT concentrations were <99th percentile there were no deaths at 30 days and only 0.8% had a MI [15]. Similarly, Dakshi et al. reported that 158 patients with unstable angina with hs-cTnT concentrations < 99th percentile had no deaths at 30 days and three (2%) MIs [16]. While prior studies investigated the incidence of unstable angina when using an hs-cTnT assay, our study is the first to evaluate the incidence using an hs-cTnI. Evaluation of both hs-cTnI and hs-cTnT is important in this situation because some differences have been noted between these assays [17]. It is important to consider, however, that in patients with underlying CAD who did not undergo cardiac catheterization in the trial (as part of the rule-out arm, or based on the clinical decision making of the treatment team), events may have occurred beyond the 30-day mark and are not reported in this study. That being said, one of the primary incentives for enhancing the detection of UA is to limit 30-day outcomes including readmission and MACE.

Third, a significant number of patients with unstable angina in this study had angiographically unremarkable or non-obstructive disease (14 patients, 23%). Although this may seem surprising other studies have shown similar results. In the study by Paiva et al., 47% of patients with unstable angina had non-obstructive coronary disease on catheterization [18]. Also, it is well-established that coronary vasospasm can present as unstable angina [19]. In addition, it is now well-known that 6–8% of patients with MI have myocardial infarction with non-obstructive coronary arteries (MINOCA) [20]. It is possible within the overall ACS arena that this is more common in unstable angina. What remains unclear, however, is the optimal treatment strategy for such patients, which is largely driven by the work-up performed to identify the causes of MINOCA.

### Limitations

There are several limitations worth considering when analyzing the results of this study. First and foremost, this was a subgroup analysis of the main RACE-IT trial, and thus the endpoints discussed in this study were not assessed at the time of patient enrollment/treatment. Secondly, not all patients with ischemic ECGs were adjudicated. Only patients who went onto coronary angiography, had an elevation of hs-cTnI > 18 ng/L after ED presentation, or type 1 MI or cardiac death at 30 days were evaluated. This introduces the possibility of selection bias, as a significant number of patients in the rule-out arm of the initial RACE-IT trial were not analyzed in this study, leading to an artificially low number of patients with UA. However, we defined UA according to the ISCHEMIA trial definitions, requiring accelerating ischemic symptoms with troponin values less than the 99th percentile, and ECG changes consistent with MI or a diagnostic catheterization result. The number of patients meeting this set of criteria but who were placed in the rule-out arm and discharged from the ED during the trial period would be exceptionally low, and so we believe that those patients who ultimately developed a troponin elevation above the 99th percentile, or underwent a cardiac catheterization, were included in the adjudication process. Similarly, results of cardiac catheterization are reported only for patients who underwent the procedure based on clinical decision making during the trial period, introducing the possibility of verification bias. Also, treatment of patients presenting to the ED enrolled in the primary RACE-IT trial was left to the discretion of the treating physicians; therefore as a subgroup analysis this study had no control over who went to catheterization and who did not. A significant amount of clinical decision-making factors into the treatment of such patients, and this is acknowledged by the research team. Similarly, downstream testing and decision making by the treatment team may have been impacted by the trial protocol/patient enrollment, depending on which group they were enrolled in or the time period of the trial. Lastly, it is important to consider the racial disparities between our trial population, which is largely reflective of the Metropolitan Detroit area, and various regions across the United States and abroad. The results of this prespecified secondary analysis should be extrapolated with caution.

## 5. Conclusions

Unstable angina is rare in patients with low hs-cTnI values at presentation to the ED and these patients have an excellent 30-day prognosis. These patients tend to not have high-risk anatomy and a significant number have non-obstructive disease or angiographically unremarkable coronary arteries.

## Figures and Tables

**Table 1:** Baseline characteristics.

	Unstable Angina	No Unstable Angina	*p* Value
	*n* = 60	*n* = 32,551	
Demographics			
Age—years	63 [57–74]	59 [45–71]	**0.015**
Sex			**0.005**
Female	22 (37%)	18,683 (57%)	
Male	38 (63%)	13,861 (43%)	
Race			0.2
White	42 (70%)	19.374 (60%)	
Black	12 (20%)	9380 (29%)	
Medical History			
Hypertension	48 (80%)	15,555 (48%)	**<0.001**
Diabetes	20 (33%)	7077 (22%)	**0.03**
Coronary artery disease	32 (53%)	3596 (11%)	**<0.001**
Peripheral vascular disease	6 (10%)	1323 (4.1%)	**0.035**
Congestive heart failure	8 (13%)	3494 (11%)	0.5
Atrial fibrillation	6 (10%)	2886 (8.9%)	0.8
Chronic lung disease	12 (20%)	6680 (21%)	>0.9
Baseline Cr—mg/dL	0.97 [0.84–1.10]	0.88 [0.72–1.08]	**0.018**
Baseline GFR—mL/min	77 [61–92]	83 [63–100]	**0.030**

Cr = creatinine; GFR = glomerular filtration rate. Variables represented as median with interquartile range [IQR] or number (*n*) with percentage (%). Significant *p* values bolded.

**Table 2:** Coronary angiography in unstable angina patients.

	*n* = 60
No angiographic disease	7 (12%)
Non-obstructive CAD	7 (12%)
Patent stents/grafts	3 (5.0%)
Obstructive CAD ^+^	46 (77%)
Single vessel	30 (50%)
Two vessels	9 (15%)
Three vessels	7 (12%)
Left main involvement	2 (3.3%)
Occluded graft(s)	2 (3.3%)

CAD = coronary artery disease. Variables are represented as number (*n*) with percentage (%). ^+^ Does not include epicardial vessels with patent stents, or patent bypass grafts.

**Table 3:** ECG results in Unstable Angina Patients.

	*n* = 60
Rhythm	
Normal sinus rhythm	33 (55%)
Sinus bradycardia	2 (3.3%)
RBBB	3 (5.0%)
Ischemic changes ^+^	17 (28%)
TWI	15 (25%)
Anterior	7 (12%)
Lateral	10 (17%)
Inferior	3 (5.0%)
ST segment depression	3 (5.0%)
Non-specific ST segment changes	5 (8.3%)

ECG = electrocardiogram; RBBB = right bundle branch block; TWI = t-wave inversion; CAD = coronary artery disease. Variables represented as number (*n*) with percentage (%). ^+^ According to definitions of ISCHEMIA trial (see Section 2).

## Data Availability

The raw data supporting the conclusions of this article will be made available by the authors on request.

## Abbreviations

CK-MB	creatinine kinase
CAD	coronary artery disease
ECG	electrocardiogram
ED	emergency department
Hs-cTnI	high-sensitivity cardiac troponin I
MACE	major adverse cardiovascular event
MI	myocardial infarction

## References

[1] Thygesen K., Alpert J.S., Jaffe A.S., Chaitman B.R., Bax J.J., Morrow D.A., White H.D., Executive Group on behalf of the Joint European Society of Cardiology (ESC), American College of Cardiology (ACC), American Heart Association (AHA) (2018). Fourth universal definition of myocardial infarction (2018). J. Am. Coll. Cardiol..

[2] Gulati M., Levy P.D., Mukherjee D., Amsterdam E., Bhatt D.L., Birtcher K.K., Blankstein R., Boyd J., Bullock-Palmer R.P., Writing Committee Members (2021). 2021 AHA/ACC/ASE/CHEST/SAEM/SCCT/SCMR guideline for the evaluation and diagnosis of chest pain: A report of the American College of Cardiology/American Heart Association Joint Committee on Clinical Practice Guidelines. J. Am. Coll. Cardiol..

[3] Braunwald E., Morrow D.A. (2013). Unstable angina: Is it time for a requiem?. Circulation.

[4] Mendis S., Thygesen K., Kuulasmaa K., Giampaoli S., Mahonen M., Blackett K.N., Lisheng L. (2011). World Health Organization definition of myocardial infarction: 2008–09 revision. Int. J. Epidemiol..

[5] Antman E.M., Tanasijevic M.J., Thompson B., Schactman M., McCabe C.H., Cannon C.P., Fischer G.A., Fung A.Y., Thompson C., Wybenga D. (1996). Cardiac-specific troponin I levels to predict the risk of mortality in patients with acute coronary syndromes. N. Engl. J. Med..

[6] Braunwald E., Antman E.M., Beasley J.W., Califf R.M., Cheitlin M.D., Hochman J.S., Jones R.H., Kereiakes D., Kupersmith J., Levin T.N. (2000). ACC/AHA guidelines for the management of patients with unstable angina and non-ST-segment elevation myocardial infarction: A report of the American College of Cardiology/American Heart Association Task Force on Practice Guidelines (Committee on the Management of Patients with Unstable Angina). J. Am. Coll. Cardiol..

[7] Lucci C., Cosentino N., Marenzi G. (2023). Unstable angina: A clinical entity on the verge of extinction?. Int. J. Cardiol..

[8] Miller J., Cook B., Gandolfo C., Mills N.L., Mahler S., Levy P., Parikh S., Krupp S., Nour K., Klausner H. (2024). Rapid Acute Coronary Syndrome Evaluation Over One Hour with High-Sensitivity Cardiac Troponin I: A United States-Based Stepped-Wedge, Randomized Trial. Ann. Emerg. Med..

[9] Miller J., Cook B., Singh-Kucukarslan G., Tang A., Danagoulian S., Heath G., Khalifa Z., Levy P., Mahler S.A., Mills N. (2021). RACE-IT—Rapid Acute Coronary Syndrome Exclusion using the Beckman Coulter Access high-sensitivity cardiac troponin I: A stepped-wedge cluster randomized trial. Contemp. Clin. Trials Commun..

[10] Christenson R.H., Duh S.H., Mullins K.E., LeClair M.M., Grigorov I.L., Peacock W.F. (2020). Analytical and clinical characterization of a novel high-sensitivity cardiac troponin assay in a United States population. Clin. Biochem..

[11] Maron D.J., Hochman J.S., Reynolds H.R., Bangalore S., O’Brien S.M., Boden W.E., Chaitman B.R., Senior R., López-Sendón J., Alexander K.P. (2020). Initial Invasive or Conservative Strategy for Stable Coronary Disease. N. Engl. J. Med..

[12] Thygesen K., Alpert J.S., Jaffe A.S., Chaitman B.R., Bax J.J., Morrow D.A., White H.D., Executive Group on behalf of the Joint European Society of Cardiology (ESC)/American College of Cardiology (ACC)/American Heart Association (AHA)/World Heart Federation (WHF) Task Force for the Universal Definition of Myocardial Infarction (2018). Fourth Universal Definition of Myocardial Infarction (2018). Circulation.

[13] Olubowale O.T., Safford M.M., Brown T.M., Durant R.W., Howard V.J., Gamboa C., Glasser S.P., Rhodes J.D., Levitan E.B. (2017). Comparison of Expert Adjudicated Coronary Heart Disease and Cardiovascular Disease Mortality with the National Death Index: Results from the REasons for Geographic and Racial Differences in Stroke (REGARDS) Study. J. Am. Heart Assoc..

[14] Wilson S.R., Sabatine M.S., Braunwald E., Sloan S., Murphy S.A., Morrow D.A. (2009). Detection of myocardial injury in patients with unstable angina using a novel nanoparticle cardiac troponin I assay: Observations from the PROTECT-TIMI 30 Trial. Am. Heart J..

[15] Gallone G., Magnoni M., Vergani V., Ceriotti F., Angeloni G., Scarano P., Maseri A., Cianflone D. (2020). Short-term prognosis of unstable angina in the era of high-sensitivity cardiac troponin: Insights for early rule-out strategies. Coron. Artery Dis..

[16] Dakshi A., Salmon T., Collinson P., Ihsan J., Campbell M., Khand A. (2023). Unstable angina in the context of high-sensitive troponins: Still a marker of high risk? A comparison of outcomes with adjudicated type 1 myocardial infarction. Int. J. Cardiol..

[17] Rubini Gimenez M., Twerenbold R., Reichlin T., Wildi K., Haaf P., Schaefer M., Zellweger C., Moehring B., Stallone F., Sou S.M. (2014). Direct comparison of high-sensitivity-cardiac troponin I vs. T for the early diagnosis of acute myocardial infarction. Eur. Heart J..

[18] Paiva L., Vieira M.J., Baptista R., Ferreira M.J., Gonçalves L. (2024). Unstable Angina: Risk Stratification for Significant Coronary Artery Disease in The Era of High-Sensitivity Cardiac Troponin. Glob. Heart.

[19] Manolis A.S., Manolis A.A., Manolis T.A., Melita H. (2018). Acute coronary syndromes in patients with angiographically normal or near normal (non-obstructive) coronary arteries. Trends Cardiovasc. Med..

[20] Parwani P., Kang N., Safaeipour M., Mamas M.A., Wei J., Gulati M., Naidu S.S., Merz N.B. (2023). Contemporary Diagnosis and Management of Patients with MINOCA. Curr. Cardiol. Rep..

